# Use of Cognitive Aids: Results from a National Survey among Anaesthesia Providers in France and Canada

**DOI:** 10.1155/2020/1346051

**Published:** 2020-05-06

**Authors:** Antonia Blanié, Matthieu Kurrek, Sophie Gorse, Dimitri Baudrier, Dan Benhamou

**Affiliations:** ^1^Centre de simulation LabForSIMS, Faculté de médecine Paris Saclay, 94275 Le Kremlin Bicêtre, France; ^2^Département d'Anesthésie-Réanimation, CHU Bicêtre, 94275 Le Kremlin Bicêtre, France; ^3^Comité Analyse et Maîtrise du Risque, Société Française d'Anesthésie et de Réanimation (SFAR), Paris, France; ^4^Département d'Anesthésie-Réanimation, CHU Toulouse, France et University of Toronto, Toronto, Canada

## Abstract

**Introduction:**

The use of cognitive aids (CAs) during critical events is thought to be useful. However, whether CAs are known and used by French and Canadian anaesthesia providers is not clear.

**Methods:**

A survey was emailed to French and Canadian anaesthesia providers in 2017 through their respective national societies. It consisted of 23 questions about the participants' demographics and their knowledge, use, and impact of CAs. A second survey was sent to French simulation centres.

**Results:**

912 responses were recorded in France and 278 in Canada (overall response rate: 7% and 11%, respectively). Among the respondents, 700/899 in France (78%) versus 249/273 (91%) in Canada were familiar with the concept of cognitive dysfunction during a crisis and 501/893 (56%) in France versus 250/271 (92%) in Canada knew the concept of CAs. Amongst those respondents who knew about CAs, 189/492 (38%) in France versus 108/244 (44%) in Canada stated that they had already used a CA in real life and 225/493 (45%) in France versus 126/245 (51%) in Canada had received training in their use. Simulation was the principal modality for training in 150/225 (67%) of cases in France versus 47/126 (37%) in Canada. Among the 28/50 French simulation centres which responded (2018 January), 27 organised sessions in anaesthesia and 22 used CAs.

**Conclusion:**

CAs were better known in Canada than in France, but their actual use in real life was low in both countries. Simulation appears to play a potentially important role training anaesthesia providers in the use of CAs.

## 1. Introduction

Critical events are fortunately very rare, but if they occur then adherence to best practice guidelines will contribute to optimal outcomes. However, good memory and clinical judgment may both deteriorate due to the increased stress and heavy mental workload and, under those circumstances, deviations from clinical guidelines are very frequent (46.5%) [1, 2]. Applying a concept already used in aviation, Gaba created the notion of crisis resource management in anaesthesia [3] and is one of the first researchers to show the potential benefits of working with cognitive aids (CAs) [4]. CAs (also named critical event checklists, sometimes grouped together in the form of an emergency manual) are aimed to increase clinical performance by guiding clinical reasoning and by inviting to implement life-saving actions [5, 6]. During critical event simulations, their use decreased the percentage of omission errors by 73% in 106 operating room scenarios [6]. The potential usefulness of CAs has been demonstrated in several publications, and their widespread use has been advocated [5, 7–9]. Teams both at Harvard and Stanford published CAs for anaesthesia providers on their respective websites [10–13].

While the safety culture and use of CAs during critical events has been emphasized in English publications for at least two decades [5, 13], the degree of familiarity with CAs and their adoption in France are not clear. Since 2016, several initiatives in France began to investigate and create anaesthesia-related CAs (written in French), most notably the French Society of Anaesthesia and Intensive Care Medicine (SFAR) [14], the latter of which are now freely available on its website and have a corresponding free smartphone application. Because the introduction of these safety measures has been more recent in France, we hypothesised that the knowledge and use of CAs by anaesthesia providers are poor when compared to Canada.

The objective of this study was to assess the knowledge and use of CAs by anaesthesia providers in France and Canada in 2017 by using a common electronic questionnaire.

## 2. Materials and Methods

### 2.1. Study Design

This prospective study was approved by the ethics committee of the SFAR in France (IRB 00010254-2017-035). It was reviewed by the Co-Chair of the Ethics Board of the Scarborough and Rouge Hospital in Toronto, Canada, and considered to be a quality improvement project and exempt from approval for Canada.

The questionnaire was created using a commercial online platform (Survey Monkey®) by three academic anaesthesia providers with experience in CAs (AB, DB, and MK). It included 23 questions about the participants' characteristics, knowledge, use, and impact of CAs (Appendix 1). This questionnaire was first reviewed and tested and then approved by SFAR (Committee on Risk Management) and SoFraSimS (French Society of Simulation in Healthcare).

The questionnaire was distributed in the Fall of 2017 via email by the SFAR (in France) and the CAS (Canadian Anesthesiologists' Society in Canada) using their respective member emailing lists (in France: 13,400 members; in Canada: 2,485 members). A reminder email message was sent three weeks after the first email message.

The second part of the study was an online French survey sent in 2018 January to the 50 simulation centres registered in the SoFraSimS database. The questionnaire was created by experts in CAs and simulation (AB, DB, and DBa) and included 17 questions about centre characteristics, whether CAs are being used in these centres and if so, how they are being implemented. This questionnaire was also officially approved by the SFAR (Committee on Risk Management) and SoFraSimS (French Society of Simulation in Healthcare).

### 2.2. Statistical Analysis

Descriptive results are expressed using means or percentages. Formal statistical comparison was not performed between Canadian and French responses.

## 3. Results and Discussion

### 3.1. Results

#### 3.1.1. Respondents' Demographics

After the initial and reminder messages had been sent, 912 responses were recorded in France (overall response rate: 7%) and 278 responses in Canada (overall response rate: 11%). The majority of participants were anaesthetists with at least 5 years experience after graduation (in France, *n* = 509/912 (56%) and in Canada *n* = 207/278 (74%)). The population characteristics of French and Canadian respondents are shown in Table 1. In some cases, questionnaires were incompletely filled out, explaining why the denominator may be different from one question to another.

#### 3.1.2. Knowledge of Cognitive Dysfunction and CAs

In France, 700/899 of the respondents (78%) were aware of the notion of cognitive dysfunction (forgetting steps that are ordinarily well known) during a critical event and 641/898 (71%) stated that they had already been victim of this in their working life. In Canada, 249/273 (91%) knew this notion and 216/273 (79%) had already encountered this situation in their clinical practice.

The concept of CAs was known by 501/893 (56%) of the French respondents, of whom 332/501 (66%) indicated of having CAs available in their workplace, whereas 250/271 (92%) of Canadian respondents knew this concept, of whom 212/250 (85%) indicated of having them available in their workplace.

Among respondents who had knowledge of CAs in France, 221/496 (45%) knew at least one institution that had published one: the most widely known were those published by the SFAR (*n* = 188/220, 86%), MAPAR (*n* = 99/220, 45%), Stanford University (*n* = 92/220, 42%), Harvard University (*n* = 20/220, 9%), and others (*n* = 28/220, 13%). In Canada, 68% knew an example of an institution that had published a CA (*n* = 168/248) and the most widely known were the CAs from Stanford (79%, *n* = 132/220) and Harvard (41%, *n* = 69/220).

#### 3.1.3. Practical Use of CA in Real Life

Among the 501 French and the 250 Canadian respondents who had some knowledge of CAs, the use of CAs in hospital practice is described in Table 2. Among all the survey respondents, the professional impact of this survey is described in Table 3.

#### 3.1.4. Formal Training on the Use of CA

Among respondents who had some knowledge of CAs, 225/493 (45%) in France and 126/245 (51%) in Canada had received formal training on how to use them. Among the healthcare professionals who had CAs available at the workplace, 77/330 (23%) in France and 57/211 (27%) in Canada had been trained in their use prior to implementation.

Education was mainly through simulation in 150/225 of responses (67%) in France and 47/126 (37%) in Canada. Regarding simulation-based education, among respondents who knew CAs, 149/489 (30%) had used them during these sessions in France and 115/243 (47%) in Canada, respectively. The description of use during simulation sessions is recorded in Table 4.

#### 3.1.5. Use of CA in Simulation according to French Simulation Centres Responses

In the online survey of French simulation centres (January 2018), 28/50 (56%) centres responded and 27 organised high-fidelity simulation sessions in anaesthesia. The characteristics of responding centres are described in Table 5. More than three-quarters of centres used CAs in their high-fidelity simulation sessions in anaesthesia (*n* = 22/27, 82%). CAs were presented in 22/22 (100%) of the centres during debriefing, while 8/22 (36%) presented them during prebriefing (introduction of the session). In few centres, CAs were sent to participants before the sessions (*n* = 2/22, 9%). The use of CAs was set as a learning objective during the debriefing in 16/18 (89%) of the centres. The paper format was used by 22/22 (100%) of centres and 6/22 (27%) of centres used also electronic CAs. The most common CAs used were CAs from SFAR (19/22, 86%) and locally developed CAs (10/22, 46%) (several possible responses). The scenarios for which CAs were most commonly used were (in decreasing order) as follows: malignant hyperthermia (89%), cardiac arrest (78%), local anaesthetic systemic toxicity (78%), and postpartum haemorrhage (67%).

## 4. Discussion

In 2017, the majority of French and Canadian anaesthesia providers responding to the survey knew the concept of cognitive dysfunction during a crisis (78% and 91%, respectively) and 56% of respondents in France and 92% in Canada were aware of the concept and availability of CAs. However, only about 40% of those who know about CAs had already used them in real life and only about 50% had been trained in their use (in France, mainly through simulation training sessions). The perceived usefulness of CAs was strong both in France and Canada.

The majority of Canadian responders (92%) knew CAs as compared to only half of responders in France (56%), probably because of the close link between Canadian anaesthetists and the United States, where the CAs have gained significant traction over the past 10 years. The French results seem similar to those published 15 years ago in the United States when in 2004, prior to the start of widespread promotion of CAs, a US survey on the use of a cardiac arrest checklist found that 59% of professionals surveyed had heard of cognitive aids, but only 12% had used them at least once [15].

Among participants aware of the concept of CAs, only 38% and 44% had already used them in real life in France and Canada, respectively. This difference between knowledge and actual use confirms the well-known difficulties of implementing new strategies [13, 16]. In a simulation-based study, although anaesthesia trainees had already been exposed to CAs and had a favourable opinion in clinical practice, almost one-third assigned to the use of CAs did not use them [17]. In a 2016 study performed in Stanford, Goldhaber-Fiebert et al. reported that only 45% of respondents had used CAs at least once in real life after their implementation [12]. Thus, even with participants who had already been sensitized and encouraged to use CAs, their use is not systematic during crisis [18]. In France, the development of CAs is continuing to grow since they were first made available (in French) in 2016 by the French Society of Anaesthesia and Intensive Care Medicine (SFAR) [14, 19]. The number of presentations at meetings and French publications about CAs is also increasing [20].

In our survey, we found that paper was the most frequently used format for CAs both in France and Canada, and this is in line with the preferences of the participants during a recent study [17]. At the time of the survey, only one institution in France had made CAs available electronically, as a smartphone App (MAPAR). The optimal format for CAs remains unknown, and the use of CAs in electronic format may increase as the SFAR has recently released its smartphone App (ACAR Android® et iOS®).

In our study, among participants aware of CAs (regardless of availability at work or not), only half had been formally trained in France and Canada. And when CAs are available in the workplace, only a quarter have been trained before their implementation. This is a very important finding as unfamiliarity with a CA has been shown to lead to suboptimal care. In a study published in 2017, Everett et al. showed that, during an unexpected serious event in an operating room such as a cardiac arrest, the use of a crisis checklist by a multidisciplinary team did not improve medical management of the event or the nontechnical skills of the participants [21]. It was noted that no prior training for their use had been provided to participants (and the correct checklist was only used in 75% of cases). This and other studies highlight the need to adequately train professionals to make CAs an effective tool as part of the implementation strategy [21, 22]. In our study, the training tool for CAs use was simulation in the majority of cases in France although this was less often the case in Canada. While the use during simulation sessions was frequent, their use was inconsistent, as it was mainly just discussed in the debriefing—in 79% of the cases. In the online survey of French simulation centres, more than three-quarters of centres stated that they use CAs in their high-fidelity simulation sessions in anaesthesia. Simulation is indeed a powerful tool to practice and learn management of critical events and the use of CAs. The access to a simulation centre, however, remains a challenge for the majority of healthcare practitioners, and this will continue to be the focus of efforts in continuing education both in France and Canada.

Respondents to the survey uniformly expressed great interest in the use of CAs. In France, this survey stimulated the interest of respondents in CAs when they did not know them yet. This survey itself served, therefore, as a method to spread knowledge on CAs and might contribute to the implementation process [13, 16, 23].

The main limitation of this study is the low response rate (in France 7% and in Canada 11%), which is the main reason for not performing any statistical analysis in our sample. An additional concern is related to the different professional practice between the two countries. Nurse anaesthetists are well represented in French hospital practice, whereas this profession does not exist in Canada. The response rate was, however, in line with the published median response rate to electronic surveys of approximately 16% [24]. In addition, most of our results support the initial hypothesis. For example, a larger number of Canadian respondents were aware of the notion of cognitive dysfunction (91 versus 78%), more respondents from Canada were aware of a published cognitive aid model (68 versus 48%), and a larger number of Canadian respondents had access to CAs in their hospital practice (85 versus 66%).

We also cannot exclude a selection bias as some respondents may have taken the time to complete the questionnaire because they knew and had some interest in using CAs. This phenomenon may thus cause an overestimation of the number of physicians who knew CAs in our project. There are few large-scale studies in the literature on knowledge and use of CAs [18]. Even if overestimated, the results show that knowledge and use are still modest.

As described above, the implementation process for CAs is complex and requires a combination of strategies [13, 16, 23]. Four key points to facilitate CAs implementation have been suggested: create, familiarise, use, and integrate [13]. These steps are necessary to not just implement but also to sustain effective use. Moreover, better implementation of CAs is significantly associated with better leadership and better communication in the team [25]. The factors associated with unsuccessful implementation of CAs are a large operating theatre, the lack of a “champion” in the team carrying the project and stimulating colleagues, a lack of institutional commitment, and a lack of time dedicated to training [25]. These barriers could explain in part why more widespread use of CAs as a daily tool has not yet occurred in medicine, even though patient safety might depend on it.

## 5. Conclusion

Anaesthesia providers in France and Canada were familiar with the notion of cognitive dysfunction during crisis. However, the actual use of CA was infrequent despite the strong perceived interest in both countries.

## Figures and Tables

**Table 1:** Population characteristics of the 911 French and 278 Canadian respondents.

		France, *n* (%)	Canada, *n* (%)
Profession	Anaesthetist (>5 years postgraduation)	509 (56%)	207 (74%)
	Anaesthetist (≤5 years postgraduation)	128 (14%)	31 (11%)
	Anaesthesia resident	86 (9%)	31 (11%)
	Nurse anaesthetist (student or postgraduation)	134 (15%)	0
	Others	55 (6%)	9 (3%)
Type of hospital	Public hospital (France)/university hospital (Canada)	664 (73%)	164 (59%)
	Private hospital (France)/community hospital (Canada)	245 (27%)	105 (38%)
	Others or N/A	9 (1%)	8 (3%)
Work setting	Emergencies frequently encountered (operating room or ICU, night calls) (yes)	791 (88%)	253 (92%)

N/A: not applicable.

**Table 2:** The use of cognitive aids (CAs) in hospital practice among the 501 French and the 250 Canadian respondents who had some knowledge of CAs.

	France, *n* (%)	Canada, *n* (%)
*Have access to CAs in hospital practice*	332/501 (66)	212/250 (85%)
If yes, format of CA (several possible responses)	241/330 (73%)	159/211 (78%)
Paper format in a manual at the bedside	172/330 (52%)	121/211 (57%)
Paper format in the emergency kit	137/330 (42%)	54/211 (26%)
Electronic format on the computer screen	69/330 (21%)	94/211 (45%)
Electronic access via a smartphone or a touchpad	189/492 (38%)	108/244 (45%)
*Have already used a CA at least once in practice*	Anaphylaxis	Cardiac arrest
If yes, which main clinical scenarios (several possible responses)	88/188 (47%)	54/107 (50%)
	PPH	Anaphylaxis
	84/188 (45%)	24/107 (22%)
	LAST	PPH
	49/188 (26%)	24/107 (22%)

LAST: local anaesthetic systemic toxicity; PPH: postpartum haemorrhage.

**Table 3:** Professional impact of this survey on all respondents (832 responses in France and 240 in Canada).

	France, *n* (%)	Canada, *n* (%)
Did not know CA but will use them	287/832 (35 %)	81/240 (34%)
CA known and used but will use them more often	295/832 (36%)	130/240 (54%)
CA known and not used but will use them	250/832 (30%)	81/240 (34%)

**Table 4:** Cognitive aids (CAs) use during simulation sessions among the French and the Canadian respondents who had some knowledge of CAs and used them in simulation (*n* = 149 in France and *n* = 115 in Canada).

Questions (I know CA and I used them in simulation sessions)	France, *n* = 149	Canada, *n* = 115
*How the CAs were presented in simulation (several possible responses):*		
Mainly discussed in briefing (introduction)	46/150 (31%)	64/115 (56%)
Mainly discussed in debriefing	118/150 (77%)	89/115 (77%)
CAs distributed at the end of the session	54/150 (36%)	24/115 (20%)
*How the CAs were use during critical events in simulation (several possible responses):*		
I prompted their use	64/147 (43%)	57/114 (50%)
Someone other than me prompted their use	20/147 (14%)	26/114 (23%)
We used the CAs because it was easily accessible and visible in the emergency kit	68/147 (46%)	50/114 (44%)
We assigned one team member the task of reading the steps listed in the CAs loudly	35/147 (24%)	51/114 (45%)
It was the team leader him/herself who read the steps listed in the CAs loudly to guide the team	46/147 (31%)	19/114 (17%)
I think that use of CAs improved patient care and teamwork performance	137/147 (94%)	110/113 (97%)

**Table 5:** Characteristics of French simulation centres using anaesthesia high-fidelity sessions (second survey) (*n* = 27).

Responses, *n* (%)	French simulation centres using anaesthesia session (*n* = 27)
*Number of anaesthesia high-fidelity sessions per year*	
>25/year	15/27 (56%)
11–25	7/27 (26%)
<10/year	5/27 (19%)
*Type of training provided*	
Students + CME	19/27 (70%)
CME only	7/27 (26%)
Students only	1/27 (4%)
*Interprofessional sessions (yes)*	26/27 (96%)

CME: continuous medical education.

## Data Availability

The datasets used and/or analyzed during the current study are available from the corresponding author on reasonable request.

## Supplementary Materials

Supplementary Materials Appendix 1: Questionnaire about Cognitive Aids.
