# Supplementary material for: Use of Cognitive Aids: Results from a National Survey among Anaesthesia Providers in France and Canada
Source: Anesthesiol Res Pract. 2020 May 6;2020:1346051. doi: 10.1155/2020/1346051 (PMC7225858; doi:10.1155/2020/1346051)
Supplement: Supplementary Materials — Appendix 1: Questionnaire about Cognitive Aids. [file 1346051.f1.docx]

**Appendix 1 : Questionnaire about Cognitive Aids**

**Q1- What is your profession (or level of training)?**

1. Anaesthetist (> 5 years post-graduation)
2. Anaesthetist ($\leq$5 years post-graduation)
3. Anaesthesia resident
4. Nurse anaesthetist (student or post-graduation) (France)
5. Other

**Q2- What is your primary work setting?**

1. Public Hospital (France) / University Hospital (Canada)
2. Private Hospital (France) Community Hospital (Canada)
3. Other (please specify):

**Q3- In which province do you primarily work?**

**Q4 Do you work in a setting where emergencies are very frequent (oncall, major surgeries etc.)?**

1. Yes, mostly in the Operating Room
2. Yes, mostly in the ICU
3. No

**Q5- Do you know the concept of ‘cognitive dysfunction’ (forgetting steps that are ordinarily well known) that can happen during critical events?**

1. No
2. Yes

**Q6- Has this ever happened to you?**

1. No
2. Yes

**Q7- Are you already familiar with the concept of using Cognitive Aids during critical events?**

1. No
2. Yes

**Q8- Do you have access to Cognitive Aids for critical events at your work?**

1. No
2. Yes

**Q9- If yes (Q8), in which form are those Cognitive Aids available at your workplace? (several responses possible)**

1. Paper format directly accessible at (or near) the bedside of patient care
2. Paper format in the emergency kit/code cart
3. Electronic format on computer at (or near) the bedside of patient care
4. Electronic access via application on smartphone or tablet

**Q10- If yes (Q8), did you receive training for the use of Cognitive Aids when they were implemented at you work?**

1. No
2. Yes

**Q11- Do you know any institutions that have created Cognitive Aids?**

1. No
2. Yes

**Q12- If yes (Q11), Which ones? (several responses possible)**

1. Stanford Emergency Manual
2. Harvard Critical Event Checklist
3. Other (please specify):

**Q13- Have you already received training in the use of those Cognitive Aids? (several responses possible)**

1. No
2. Yes, in a lecture
3. Yes, in a workshop
4. Yes, during a simulation
5. Yes, at a meeting

**Q14- Have you ever used a Cognitive Aid during a real-life critical event?**

1. No, never
2. Yes

**Q15- If yes (Q14), during which situation(s)? (several responses possible)**

1. Anaphylaxis
2. Malignant Hyperthermia
3. Operating room Fire
4. Cardiac Arrest (adult)
5. Cardiac Arrest (pediatric)
6. Cardiac Arrest (pregnant patient)
7. Difficult intubation
8. Local anaesthetic systemic toxicity
9. Postpartum Haemorrhage
10. Power Failure
11. Oxygen Failure
12. Cardiac Arrest (adult) in the ICU
13. Accidental extubation in the ICU
14. Sudden desaturation in the ICU
15. Difficult intubation in the ICU
16. Sudden hypotension in the ICU
17. Other (please specify):

**Q16- Did you already have the opportunity to use a Cognitive Aid during a simulation session?**

1. No
2. Yes

**Q17- If no (Q16), why ?**

1. I don’t have any experience with simulation
2. I have already participated in a simulation session, but Cognitive Aids were not discussed or used
3. A Cognitive Aid was available but not used
4. No Cognitive Aid was available
5. I didn’t think I needed one

**Q18- If yes (Q16), please describe the use of Cognitive Aids during the simulation: (several responses possible)**

1. Their use was discussed before the simulation session (in briefing/introduction)
2. Their use was discussed during the debriefing
3. Participants were handed out the Cognitive Aids at the end of the session

**Q19- Describe the use of Cognitive Aids during real or simulated critical**

**events: (several responses possible)**

1. I prompted their use
2. Someone other than me prompted their use
3. We used the Cognitive Aid because it was easily accessible and visible in the emergency kit
4. We assigned one team member the task of reading the steps listed in the Cognitive Aid out loud
5. It was the team leader him/herself who read the steps listed in the Cognitive Aid out loud to direct the team

**Q20- If you have used a Cognitive Aid during a real or simulated critical incident, please describe their usefulness (one response only):**

1. I think their use improved patient care / performance
2. I think their use did not improve patient care / performance

**Q21- After participating in this survey, are you planning to use Cognitive Aids more often (if you use them already) or (if you have not yet used them) to implement them?**

1. No, I don’t see the interest
2. Yes

**Q22- If no (Q21), for what reasons to you think Cognitive Aids are of no interest?**

**Q23- if yes (Q21), please indicate what you are planning to change:**

1. This survey stimulated my interest in Cognitive Aids which I previously didn’t know anything about
2. I already know Cognitive Aids – I have never used them before, but I will try to use them more often from now on
3. I already know Cognitive Aids – I already use them and I will try to use them more often
